# Genes That Mediate Starch Metabolism in Developing and Germinated Barley Grain

**DOI:** 10.3389/fpls.2021.641325

**Published:** 2021-03-01

**Authors:** Helen M. Collins, Natalie S. Betts, Christoph Dockter, Oliver Berkowitz, Ilka Braumann, Jose A. Cuesta-Seijo, Birgitte Skadhauge, James Whelan, Vincent Bulone, Geoffrey B. Fincher

**Affiliations:** ^1^Australian Research Council Centre of Excellence in Plant Cell Walls, School of Agriculture, Food and Wine, University of Adelaide, Glen Osmond, SA, Australia; ^2^Carlsberg Research Laboratory, Copenhagen, Denmark; ^3^School of Life Sciences and ARC Centre of Excellence in Plant Energy Biology, La Trobe University, Bundoora, VIC, Australia; ^4^Adelaide Glycomics, School of Agriculture, Food and Wine, University of Adelaide, Glen Osmond, SA, Australia

**Keywords:** aleurone, endosperm, gene families, *Hordeum vulgare*, RNA-seq, scutellum, starch synthesis, starch degradation

## Abstract

Starch is synthesized in the endosperm of developing barley grain, where it functions as the primary source of stored carbohydrate. In germinated grain these starch reserves are hydrolyzed to small oligosaccharides and glucose, which are transported to the embryo to support the growth of the developing seedling. Some of the mobilized glucose is transiently stored as starch in the scutellum of germinated grain. These processes are crucial for early seedling vigor, which is a key determinant of crop productivity and global food security. Several starch synthases (SS), starch-branching enzymes (SBEs), and starch debranching enzymes (isoamylases, ISA), together with a limit dextrinase (LD), have been implicated in starch synthesis from nucleotide-sugar precursors. Starch synthesis occurs both in the developing endosperm and in the scutellum of germinated grain. For the complete depolymerization of starch to glucose, α-amylase (Amy), β-amylase (Bmy), isoamylase (ISA), limit dextrinase (LD), and α-glucosidase (AGL) are required. Most of these enzymes are encoded by gene families of up to 10 or more members. Here RNA-seq transcription data from isolated tissues of intact developing and germinated barley grain have allowed us to identify the most important, specific gene family members for each of these processes *in vivo* and, at the same time, we have defined in detail the spatio-temporal coordination of gene expression in different tissues of the grain. A transcript dataset for 81,280 genes is publicly available as a resource for investigations into other cellular and biochemical processes that occur in the developing grain from 6 days after pollination.

## Introduction

Starch reserves in plants represent one of the most important polysaccharides for human societies. Whether the source be cereal grains, tubers, nuts, fruit, vegetables, or other plants, starch constitutes a major proportion of our daily caloric intake. Starch and its chemically modified derivatives also find many industrial applications, which include their use as thickeners, sweeteners, stabilizers or gelling agents in food production, for the production of edible and biodegradable packaging films, in paper manufacture, in the pharmaceutical and cosmetic industries, for renewable liquid biofuel production and as a source of fermentable sugars for brewing industries (Smith, 2001; Tester et al., 2004; Waterschoot et al., 2015; Blennow, 2018). Starch is deposited in simple or compound granules of varying sizes and shapes in the chloroplasts of leaves and in the amyloplasts of grains and tubers. Within these granules, the major constituents of starch, namely the branched (1,4;1,6)-α-glucan amylopectin and the essentially unbranched (1,4)-α-glucan amylose, form alternating crystalline and amorphous layers (Jenkins et al., 1993). Most starches contain 70–80% amylopectin and 20–30% amylose; phospholipids and free fatty acids can be associated with the amylose and some protein is attached to the surface of the granules (Tester et al., 2004).

**FIGURE 1:**
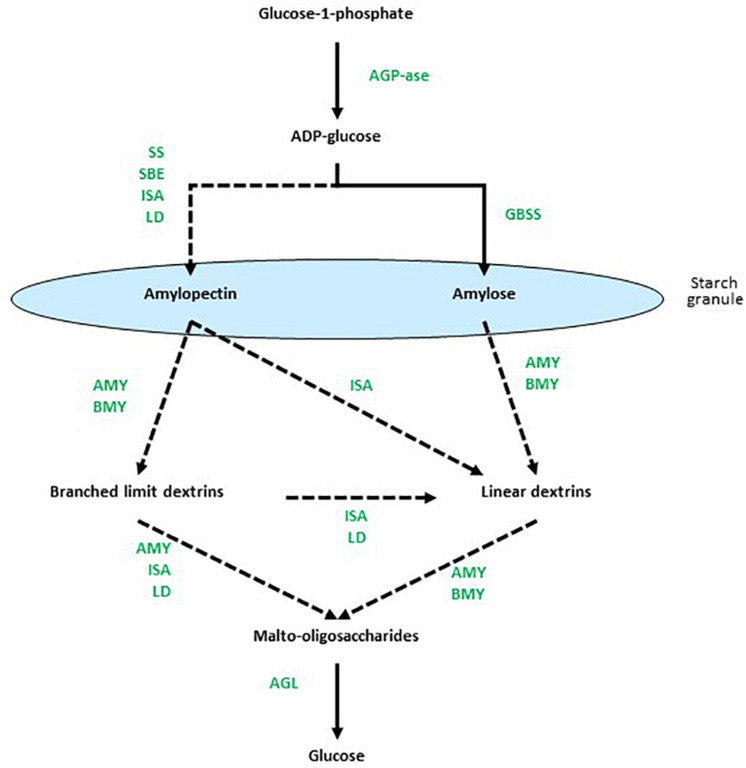
Overview of enzymes involved at different stages of starch metabolism. Dotted arrows indicate multi-enzyme processes. Based on data from Radchuk et al. (2009), Zeeman et al. (2010), Zhang and Li (2017), MacNeill et al. (2017), and Skryhan et al. (2018). AGP-ase denotes ADPG-pyrophosphorylase, SS is starch synthase, SBE is starch branching enzyme, ISA is isoamylase, GBSS is granule-bound starch synthase, AMY is α-amylase, Bmy is β-amylase, LD is limit dextrinase, and AGL is α-glucosidase.

The biosynthesis of starch is mediated primarily by multiple isoforms of starch synthases (SS) and starch-branching enzymes (SBEs) and, perhaps counterintuitively, the debranching enzyme isoamylase (ISA) is also important (Smith, 2001). In addition, limit dextrinase (LD) might play a role in starch synthesis (Martin and Smith, 1995; Burton et al., 1999). The α-glucosyl residues of starch are obtained through the conversion of sucrose to glucose 6-phosphate, which is transported across the plastid membrane, isomerized to glucose 1-phosphate and converted by the enzyme ADP-glucose pyrophosphorylase (AGP) to the nucleotide-sugar, ADP-glucose (ADPG). Alternatively, ADPG can be synthesized in the cytosol and transported into the plastid. The ADPG is the direct glucosyl donor for elongating (1,4)-α-glucan chains (Martin and Smith, 1995) and is the substrate for the SS group of enzymes. Transglycosylating SBEs introduce the (1,6)-branch points into amylose to form amylopectin. However, it was recognized many years ago that there are multiple isoforms of both the SSs and the SBEs. Thus, multiple granule-bound starch synthases (GBSSs) and several classes of soluble starch synthases (e.g., SSI, SSII, and SSIII), together with SBEs and possibly LDs, function in many interdependent combinations to synthesize starch with different amylopectin:amylose ratios and with a range of different internal chain lengths in the amylopectin component (Smith, 2001; Zhong et al., 2020). The ISA enzyme catalyses the hydrolysis of (1,6)-branch points in amylopectin, but has nevertheless been shown to participate in starch biosynthesis, where it affects the internal chain length of amylopectin (Rahman et al., 1998). The proposed roles of each of these enzyme classes are summarized in Figure 1. Subsequent phosphorylation and de-phosphorylation of starches are catalyzed by glucan water dikinases (GWD) and phosphoglucan water dikinases (PWD) (Mikkelsen et al., 2004; Kotting et al., 2005). The membrane transport requirements for synthesizing starch within plastids add additional levels of complexity to these biochemical considerations.

The depolymerization of starch is also a complex process that requires the concerted action of multiple enzymes (Figure 1), again with the added cell biological complication of the plastid envelope (Oates, 1997). The (1,4)-α-glucan endohydrolase, α-amylase (Amy), hydrolyses internal (1,4)-α-glucosyl linkages in both amylopectin and amylose, while the (1,6)-α-glucan endohydrolase ISA is required to hydrolyse the (1,6)-α-glucosyl linkages at branch points in amylopectin. The (1,4)-α-glucan exohydrolase, β-amylase (Bmy), hydrolyses penultimate (1,4)-α-glucosyl linkages from the non-reducing end of both amylopectin and amylose, to release the disaccharide maltose. However, Bmy is unable to bypass (1,6)-α-glucosyl linkages in amylopectin. The oligosaccharides released from amylopectin by Amy and Bmy, either separately or in combination, thus contain (1,6)-α-glucosyl residues and are known as limit dextrins. They require the additional action of LD to hydrolyze the remaining (1,6)-α-glucosyl linkages. Short (1,4)-α-linked linear maltodextrins released by the enzymes are hydrolyzed to glucose by α-glucosidases (AGL). As observed with the enzymes involved in starch synthesis, the hydrolases required for starch degradation are also encoded by multi-gene families (Radchuk et al., 2009).

Here, examination of the barley genome sequence (International Barley Genome Sequencing Consortium [IBGS] et al., 2012; Mascher et al., 2017) reveals that, with the exception of LD, the enzymes involved in the synthesis and degradation of starch are members of gene families of up to 11 individual genes (Supplementary Table S1). We have isolated tissues from the developing barley grain from 6 to 42 days post anthesis (DPA). RNA-seq has been used to generate transcript profiles of genes that mediate both starch synthesis and starch degradation in the developing grain. In addition, we have compared our RNA-seq transcript data from the developing grain with RNA-seq transcript profiles of individual tissues of germinated grain (Betts et al., 2020). The RNA-seq data provide a complete dataset for starch metabolism in both developing and germinated barley grain, through which specific members of the multiple and large gene families that mediate the biosynthetic and degradative pathways of starch metabolism have been identified and through which differences in transcribed genes between the different tissues of developing and germinated grain have been defined.

## Materials and Methods

### Plant Material: Developing Grain

Barley (*Hordeum vulgare* cv. Navigator) plants were grown under standard greenhouse conditions using day/night temperatures of 23°C/15°C. Navigator is an Australian malting barley cultivar. The date of anthesis for each barley head was assessed visually by the presence of free pollen on anthers. Grains were collected every 4 days from 6 to 42 days post anthesis (DPA) from the middle of the head. Each individual sample contained tissues collected from three grains. At 6 DPA and 42 DPA, whole grain tissue was analyzed. At 10 DPA, internal grain contents (embryo plus endosperm) were collected separately from external maternal tissues. From 14 to 38 DPA, the embryo was manually dissected from the grain and the remaining endosperm/maternal tissues were collected together; as much husk as possible was removed from the samples.

### RNA-Seq Analyses

For developing grain, RNA was prepared and sequenced from three replicate samples of each tissue at each time point, using the Spectrum^TM^ Plant Total RNA kit (Sigma-Aldrich, St Louis, MO, United States), as described by Betts et al. (2017).

RNA integrity was assessed using an Agilent 2200 TapeStation system (Agilent Technologies, Inc., Waldbronn, Germany). Libraries were prepared from total RNA using the TruSeq Stranded Total RNA with Ribo−Zero Plant kit according to the manufacturer’s instructions (Illumina^1^) for three biological replicates of each tissue type. Transcript abundances and count estimates (transcripts per million TPM) were determined using a k-mer index build from the representative transcript models (Mascher et al., 2017) using a k-mer length of 31 and the kallisto program (version 0.46.0) with 100 bootstraps (Bray et al., 2016). Principal component analysis (PCA) was performed using TPM values for all genes and the prcomp function in R. Hierarchical clustering was performed with the Partek Genomics software suite version 6.16 (Partek Incorporated, St. Louis, MO, United States). Functional gene categories were analyzed using the PageMan tool (Usadel et al., 2006) following gene annotation using Mercator (Lohse et al., 2014) of reference plant genome sequences, with manual curation.

Gene ontology (GO) enrichment analyses were undertaken using the “BiNGO” plugin program for Cytoscape (PMID 15972284); enrichment was considered statistically significant if a *p* < 0.05 was observed after Bonferroni correction. *In silico* gene discovery and annotation was performed as described in Betts et al. (2020).

### Microscopy

Mature and germinated grains were cut approximately in half along their longitudinal axes and fixed in 0.25% glutaraldehyde, 4% paraformaldehyde, and 4% sucrose in phosphate-buffered saline (PBS), pH 7.2. The grains were subsequently dehydrated and embedded in LR White resin, as described in Burton et al. (2011). Sections (1 μm) were cut on an ultramicrotome using a diamond knife and dried onto polylysine-coated microscope slides (Thermo Fisher Scientific). Sections of mature grain and 96 h germinated grain were treated with or without α-amylase (Megazyme *Bacillus licheniformis*; E-BLAAM) for 1 h at room temperature. Lugol’s solution (2% w/w KI, 1% w/w I_2_) was applied to the slides for 30 s and rinsed off with water. Sections were imaged using a Carl Zeiss microscope (Axio Imager M2, Zeiss, Germany) equipped with an AxioCam Mrm camera and processed using ZEN 2012 software (Carl Zeiss, North Ryde, Australia).

For scanning electron microscopy (SEM), samples were analyzed at 10 kV at Adelaide Microscopy (University of Adelaide) using a Quanta 450 Field Emission Gun Environmental SEM (FEI, Hillsboro, United States) for 24 h-germinated grain or using an XL-30 FEGSEM (Philips, Amsterdam) for 96 h-germinated grain. For environmental SEM at 24 h, grains were cut in half longitudinally and examined directly. For SEM at 96 h, grains were fixed for 16 h as above, washed in PBS/sucrose solution and dehydrated in an ascending ethanol series from 70 to 100% (v/v). Samples were dried using hexamethyldisilazane, mounted on aluminum stubs, and sputter coated with 5 nm platinum prior to examination.

### Chain Length Distribution of Amylopectin

Samples of scutellum or embryo were frozen in liquid nitrogen, ground to a powder and lyophilized. To approximately 20 mg of each powdered sample was added 900 μL 1% (w/v) SDS containing 5 mM (w/v) DTT. Samples were mixed and centrifuged (1 min, 10,000 g), the supernatant was removed and the SDS/DTT extraction process was repeated twice. The insoluble material was resuspended in 1 mL 70% ethanol, mixed, centrifuged and the supernatant was removed. This treatment was repeated twice. The powder was subsequently resuspended in 500 μL 20 mM Na_2_HPO_4_/NaH_2_PO_4_ buffer (pH 6.5) and 12.5 U lichenase (Megazyme, Ireland) was added for 90 min with mixing at 50°C to hydrolyze (1,3;1,4)-β-glucan. Samples were centrifuged, supernatants were removed and the insoluble material was washed in water three times and lyophilized.

The dried samples were resuspended in 100 μL 0.5 M NaOH, to which were added 800 μL water, 50 μL 1 M HCL, 50 μL of 200 mM NaOAc-HOAc buffer (pH 4.0), 1.4 U isoamylase (Megazyme, E-ISAMY, 1,000 U/mL) and 1.4 U pullulanase M2 (Megazyme, E-PULBL, 900 U/mL from *Bacillus licheniformis*). The mixture was incubated for 90 min at 40°C, neutralized with 60 μL 1 M Tris-HCl buffer (pH 8.5), heated to 100°C and centrifuged at 5,000 g for 1 min. The supernatants were collected, diluted fivefold with water and their chain length distributions were measured by high-performance anion-exchange chromatography with pulsed amperometric detection (HPAEC-PAD) as described in Botticella et al. (2018).

### Western Hybridization Analyses

Western blot detection of SSI and SBEI was performed essentially as described and using the same antibodies as Cuesta-Seijo et al. (2016), with an HRP-coupled goat anti-rabbit secondary antibody (Invitrogen) and Pierce ECL Plus Western Blotting Substrate (Thermo Fisher Scientific). Extracts from equal amounts of flour (approximately 100 μg) were loaded per lane.

## Results

### Transcript Data

For the developing grain, high quality RNA with an average integrity number of more than 7.0 was isolated from each tissue at each time point. The complete dataset of 81,280 gene sequences for developing grain, expressed as TPM, is shown in Supplementary Table S2. RNA-seq transcript data for germinated grain was from the same dataset described by Betts et al. (2020), in which transcripts from 33,421 genes were quantitated from the scutellum, the residual embryo and three aleurone sections (the proximal al1 section, the central al2 section and the distal al3 section) for 0–4 days after the initiation of germination.

**FIGURE 2:**
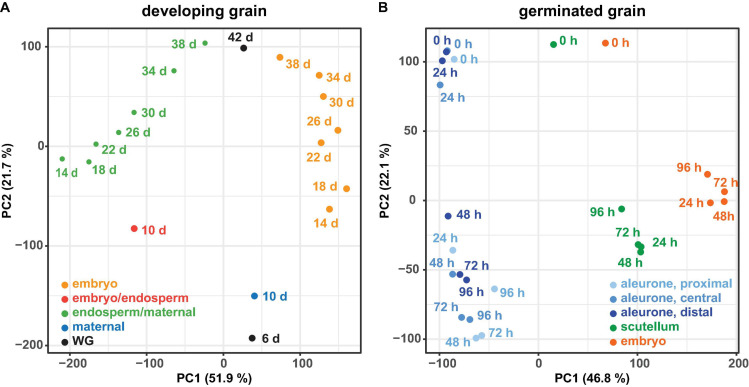
Principal component analysis of RNA-seq data. For the RNA-seq data of the developing grain **(A)** and the germinated grain **(B)** a principal component analysis (PCA) was performed using the transcripts per million (TPM) values of all genes. For both experiments the samples are separated by tissue (principal component 1, PC1) and time (principal component 2, PC2).

PCA of all genes from developing grain (Figure 2A) and germinated grain (Figure 2B) show that in each case samples are separated by tissue (principal component 1 PC1, *x*-axis) and time (PC2, *y*-axis). In developing grain, transcripts of the embryo/endosperm tissues are clearly separated from the endosperm/maternal tissues, and both also separate with time. In germinated grain, biological replicates of scutellum- and embryo-transcribed genes clustered tightly, while gene transcripts in the three aleurone tissues separated more widely with time. It should be noted that Betts et al. (2017) performed multi-dimensional scaling analyses on a similar dataset from germinated grain and also showed that samples were separated predominantly by tissue and time, respectively.

The HORVU gene identifiers (Mascher et al., 2017) for the most abundantly transcribed genes involved in starch synthesis and degradation are listed in Table 1. Starch genes were annotated using the Morex 2017 genome assembly (Mascher et al., 2017), based on comparisons with known sequences from the 2012 scaffold (International Barley Genome Sequencing Consortium [IBGS] et al., 2012), the NCBI database, and the literature (Frandsen et al., 2000; Radchuk et al., 2009; Mangelsen et al., 2011; Ma et al., 2014, 2016; Aubert et al., 2018). Where several, nearby gene models contained identical or near-identical sequences, all gene model (HORVU) identifiers are provided for completeness. A summary of updated annotations for genes encoding enzymes involved in starch synthesis and degradation is presented in Supplementary Table S1.

**TABLE 1:** Summary of most highly transcribed genes involved in starch metabolism in developing and germinated barley grain.

**Grain**	**Function**	Gene annotation*	**HORVU identifier**	**Max. TPM (time)**	Tissue**
Developing	Synthesis	*AGP-L1*	HORVU1Hr1G091600	4,608 (10 DPA)	Embryo/Endosperm
		*AGP-S2*	HORVU5Hr1G054210	896 (10 DPA)	Embryo/Endosperm
		*SBE1*	HORVU0Hr1G022810	2,747 (14 DPA)	Endosperm
		*SBE2a*	HORVU2Hr1G072500	1,382 (10 DPA)	Embryo/Endosperm
		*SBE2b*	HORVU2Hr1G077120	1,599 (10 DPA)	Embryo/Endosperm
		*GBSS1a*	HORVU7Hr1G012380	816 (10 DPA)	Embryo/Endosperm
		*GBSS1b*	HORVU2Hr1G090980	244 (34 DPA)	Embryo
		*SS1*	HORVU7Hr1G025390	828 (10 DPA)	Embryo/Endosperm
		*SS2a*	HORVU7Hr1G038420	654 (10 DPA)	Embryo/Endosperm
		*ISA1*	HORVU7Hr1G051750	300 (10 DPA)	Embryo/Endosperm
		*GWD1*	HORVU7Hr1G048330	118 (26 DPA)	Embryo
	Degradation	*AGL3*	HORVU1Hr1G057330	382 (18 DPA)	Embryo
		*Bmy1*	HORVU4Hr1G089510	8,394 (18 DPA)	Endosperm
		*PHO2*	HORVU3Hr1G085380	181 (18 DPA)	Embryo
		*PHO3*	HORVU3Hr1G085420	340 (18 DPA)	Embryo
	Modification	*DPE2*	HORVU2Hr1G021700	179 (18 DPA)	Embryo

Germinated	Synthesis	*AGP-L1*	HORVU1Hr1G091600	471 (96 h)	Scutellum
		*AGP-S2*	HORVU5Hr1G054210	116 (0 h	Aleurone al1
				311 (96 h)	Scutellum
		*ISA3*	HORVU5Hr1G070160	420 (96 h)	Scutellum
		*SBE2a*	HORVU2Hr1G072500	306 (96 h)	Scutellum
		*SS1*	HORVU7Hr1G025390	316 (96 h)	Scutellum
				102 (24 h)	Aleurone al2
		*GBSS1b*	HORVU2Hr1G090980	119 (96 h)	Scutellum
	Degradation	*AGL1*	HORVU7Hr1G106540	2,201 (72 h)	Aleurone al1
				1,619 (48 h)	Scutellum
		*Amy1_2*	HORVU6Hr1G080790	19,960 (72 h)	Aleurone al1
				3,099 (72 h)	Scutellum
		*Amy2_3*	HORVU7Hr1G091250	3,526 (72 h)	Aleurone al1
		*Bmy1*	HORVU4Hr1G089510	610 (24 h)	Aleurone al3
		*Bm*	HORVU4Hr1G084390	460 (0 h)	Scutellum
		*LD*	HORVU7Hr1G027860	6,408 (72 h)	Aleurone al1

*^∗^*AGP-S*,ADP-glucose pyrophosphorylase small subunit; *AGP-L*,ADP-glucose pyrophosphorylase large subunit; *SBE*, starch branching enzyme; *GBSS*, granule-bound starch synthase; *SS*, solublestarch synthase; *AGL*, α-glucosidase; *Bmy*,β-amylase; *PHO*, starch pyrophosphorylase; *DPE*, disproportionating enzyme; *GWD*, glucan water dikinase; *ISA*, isoamylase; *Amy*, α-amylase; *LD*, limit dextrinase. ^∗∗^As noted above for developing grain, at 10 DPA internal grain contents (embryo plus endosperm) were collected separately from external maternal tissues. From 14-38 DPA, the embryo was manually dissected from the grain and the remaining endosperm/maternal tissues were collected together (designated ‘Endosperm’ in the table above).*

### Starch Metabolism in Developing Grain

**FIGURE 3:**
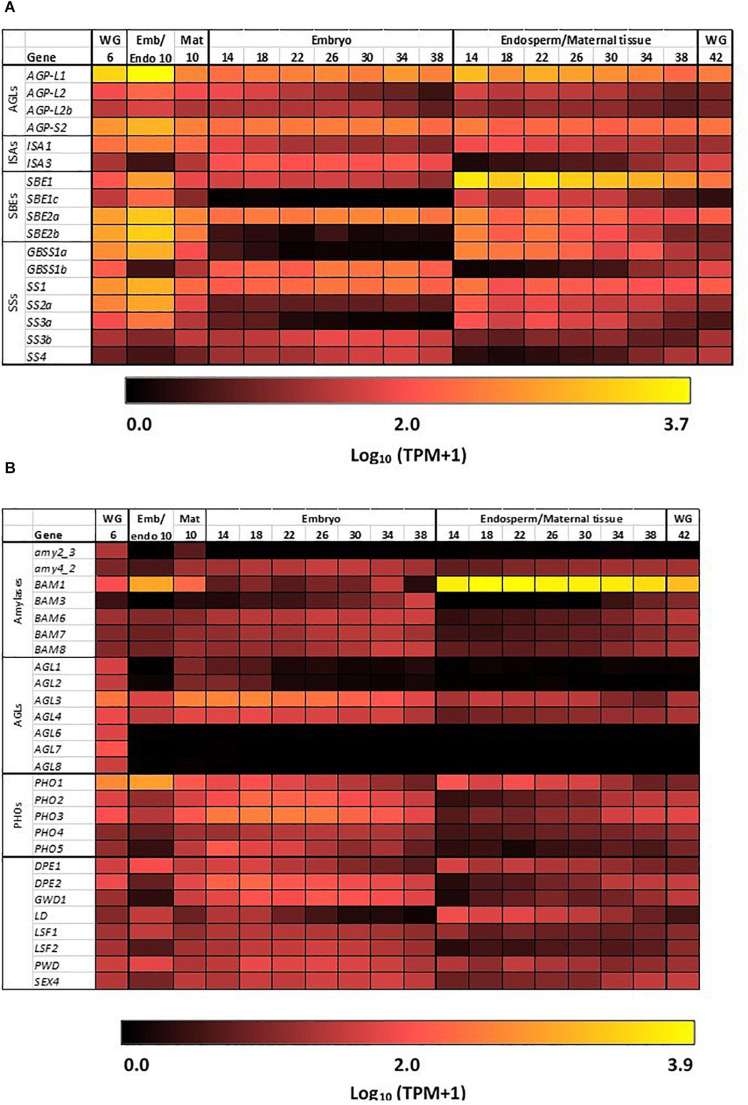
Expression heat maps of genes involved in starch metabolism in developing barley grain. **(A)** Genes involved in starch synthesis and **(B)** genes involved in starch hydrolysis. Emb, embryo and scutellum; Endo, endosperm; Mat, maternal tissue; WG, whole grain. Numbers indicate days post-anthesis. The heat map colors are log_10_ TPM: for example, a value of 1.0 (very dark red = 10 TPM; 2.0 (bright red = 100 TPM; 3.0 (orange) = 1,000 TPM, while 3.7 (yellow) is approximately 5,000 TPM.

The heat map in Figure 3A shows the most highly transcribed genes during grain development and allows the identification of important individual genes and their expression patterns over the developmental period.

The first enzyme in committing glucose to starch synthesis is AGP, which consists of two large (L) and two small (S) subunits (Stark et al., 1992). Of the four AGP subunit genes, the *AGP-L1* gene is most highly transcribed in the endosperm, where a TPM value of 4608 is observed at 10 DPA (Table 1). This value decreases steadily to 166 TPM at 38 DPA (Figure 3A and Supplementary Table S2). In the embryo, TPM values for this gene stay approximately constant in the range 195–489. Transcript levels for the *AGP-S2* gene peak at 896 TPM at 10 DPA and subsequently remain approximately constant in the range of 136–309 TPM in both the embryo and the endosperm for the duration of grain development. Much lower levels of transcripts for the *AGP-L2* and *AGP-S1* subunit genes are detected, mainly at the early time points (Supplementary Table S2). We could detect no clear correlations between the abundances of transcripts that encode the two large and two small subunits of the enzyme.

Quite distinct transcript profiles are observed for the starch synthase genes. Transcripts for the *SS1* gene, after peaking at 828 TPM at 10 DPA, are found in approximately equal abundance (range of 89–244 TPM) in both the endosperm and embryo tissues. In contrast, *SS2a* (TPM peak of 654 at 10 DPA decreasing to low levels at 38 DPA), *SS3a* (234 TPM at 10 DPA down to very low levels at 38 DPA), and *GBSS1a* (816 TPM at 10 DPA, decreasing to very low levels at 38 DPA) transcripts are found almost exclusively in the endosperm/maternal tissues. Finally, the *SS2c* (5–17 TPM), *SS3b* (23–70 TPM), and *GBSS1b* (126–244) transcripts are found mostly in the embryo (Figure 3A and Supplementary Table S2).

Transcripts of the starch branching enzyme (*SBE1*) are detected mainly in the endosperm (peak of 2747 TPM at 14 DPA, down to 462 TPM at 38 DPA). The *SBE2a* (peaking at 1382 TPM at 10 DPA in the range of about 100–400 TPM throughout) transcripts are found in both the embryo and endosperm, while transcripts of *SBE2b* (peaking at 1599 TPM at 10 DPA and decreasing to very low levels at 38 DPA) genes are transcribed mostly in the endosperm (Supplementary Table S2).

Transcripts for genes encoding the debranching enzymes ISA1 and ISA3 are detected in the developing grain (Table 1) at peak levels of 300 TPM (10 DPA) and 127 TPM (18 DPA), respectively. The *ISA1* transcripts are found in both the embryo and endosperm, while *ISA3* transcripts are found mostly in the embryo (Figure 3A and Supplementary Table S2). Transcripts for the single LD gene peaked at 78 TPM at 14 DPA in the endosperm and thereafter decreased (Supplementary Table S2). Although phosphorylation levels in cereal starches are generally low, transcripts of the α-glucan water dikinase gene (*GWD1*) were detected in the embryo of developing grain (Table 1).

In contrast to the high levels of transcripts for many starch synthetic enzymes, transcript levels for the major starch degrading enzymes are relatively low in the developing grain, as expected. Of the *Amy* genes, only transcripts for *Amy4_2* are detectable and these are predominantly in the embryo tissue extracts (Supplementary Table S2). However, very high levels of transcripts for the *Bmy1* are detected in the endosperm of developing grain, with a maximum of 8394 TPM at 18 DPA (Table 1). Transcripts for this gene are maintained at high levels from 14 DPA (5,667 TPM) until 34 DPA (5,336 TPM), and decrease slightly by 38 DPA (3,693 TPM). The high levels of transcription of this gene are also evident in the heat map shown in Figure 3B. With the exception of the *AGL3* gene, which is transcribed mainly in the embryo and maternal tissues at levels of 382 TPM at 18 DPA decreasing to 59 TPM at 38 DPA, genes encoding other enzymes involved in starch degradation are expressed at very low levels in both tissue preparations from the developing grain (Figure 3B and Supplementary Table S2).

### Starch Degradation in the Germinated Grain

**FIGURE 4:**
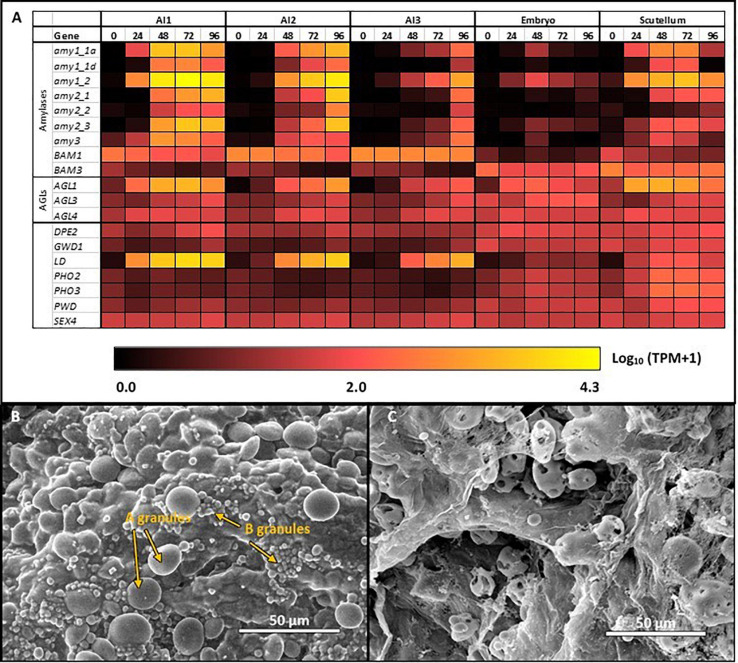
Starch degradation in the germinated grain **(A)** Expression levels of selected genes encoding starch degrading enzymes in barley tissues during germination; **(B)** SEM images of large **(A)** and small **(B)** starch granules in starchy endosperm at 24 h after the initiation of germination, before granule erosion is apparent; **(C)** SEM images of starch granules in the starchy endosperm at 96 h, when small granules have disappeared and large granules show extensive surface erosion.

The transcription patterns of the genes that mediate starch degradation in cv. Navigator are shown diagrammatically in the heat map of Figure 4A, where the different patterns of the various transcripts can be compared, together with the differences between the aleurone and scutellum tissues, and the wave of transcription from the al1 to the al3 tissues for specific *Amy*, *LD*, and *AGL* genes. The large and small starch granules of starchy endosperm cells show no obvious degradation at 24 h (Figure 4B), but at 96 h the small granules have disappeared and the large granules show extensive surface pitting and hollowing out of their cores (Figure 4C).

The most abundant transcripts in the germinated barley grain are those of the *Amy1_2* gene (Betts et al., 2020; Figure 4A). Transcript levels in the aleurone al1 cells increase from 0 to 19,960 TPM between 0 and 72 h after the initiation of germination and decrease at 96 h (Table 1 and Supplementary Table S2). The transcripts also increase to high levels in the aleurone al2 tissue, but with an approximate 24 h lag behind the al1 values. There is a further lag before the transcripts increase in the al3 tissue (Figure 4A). The *Amy1_2* gene is also transcribed in the scutellum, where transcripts peak at 3,099 TPM at 72 h (Figure 4A and Table 1).

The *Amy2_3* gene is transcribed at high levels in the aleurone, with a peak 3,526 TPM in al1 at 72 h; relatively low levels of transcripts for this gene are found in the scutellum (Figure 4A and Table 1). Other α-amylase gene transcripts include *Amy1_1a*, which peak in al1 at 72 h, and are also found in the scutellum at relatively high levels. Lower levels of transcripts for the *Amy2_1* gene (peak at 96 h in al1), the *Amy3* gene (peak at 48 h in al1), and the *Amy1_1d* gene (peak of 515 TPM at 72 h in al1) are present, predominantly in the aleurone (Figure 4A and Supplementary Table S2).

Transcripts for *Bmy1* are found right along the aleurone layer, with levels of 610 TPM at 0 h in the al3 tissue (Figure 4A and Table 1); these levels decrease with time (Figure 3A). Transcripts for the *Bmy3* gene are present in the scutellum at a level of 460 TPM at 0 h and also decrease with time (Table 1 and Figure 4A). Of the other genes that encode starch-degrading enzymes, the *LD* gene is the most highly transcribed, with levels in al1 increasing to a peak of 6,408 TPM at 72 h before dropping back at 96 h (Table 1 and Figure 4A). Transcripts for this gene are also detected at 72 h in the scutellum, but at much lower levels. The *AGL1* gene is transcribed at relatively high levels in both the aleurone and scutellum, with values peaking in al1 tissue at 2,201 TPM at 48 h and at 1,260 TPM in the scutellum at 24 h (Table 1). Following a lag of about 24 h, similar expression patterns of this gene are observed in the al2 and al3 tissue (Figure 4A).

### Starch Synthesis in the Germinated Grain

**FIGURE 5:**
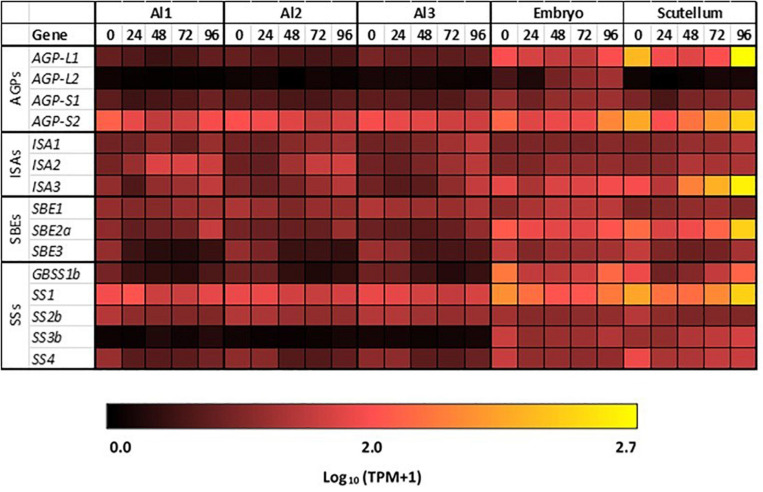
Starch synthesis during grain germination. Expression levels of genes encoding starch synthetic enzymes in barley tissues during germination. E, embryo; S, scutellum; R, rest of grain.

Transcript abundances of important genes for starch synthesis in the germinated grain are compared in the heat map shown in Figure 5, where it is evident that these transcripts are found predominantly in the scutellum; their TPM values are much lower than in the developing grain (Table 1; Betts et al., 2020). Transcripts of several genes involved in starch synthesis are detected in the germinated grain, particularly in the scutellum; note that transcripts in the remaining embryo (without the scutellum; Betts et al., 2020) of the germinated grain are not considered here. *SS1* gene transcripts increase in the scutellum to a peak of 316 TPM at 96 h (Figure 5 and Table 1). Transcripts for this *SS1* gene are also detected in the aleurone during the 96 h period, but levels are less than about 100 TPM throughout. Other genes encoding starch biosynthetic enzymes are found mainly in the scutellum, but also at lower levels in the aleurone. These include transcripts from the *AGP-L1* gene (471 TPM in the scutellum at 96 h), the *AGP-S2* gene (218 TPM at 0 h in the scutellum), the *SBE2a* gene (306 TPM at 96 h in the scutellum), the *ISA3* gene (85 TPM at 0 h rising to 420 TPM at 96 h), the *GBSS1b* gene (119 TPM at 96 h in the scutellum), and the *SS4* gene (119 TPM at 96 h in the scutellum) (Figure 5 and Supplementary Table S2).

To check whether full length enzymes encoded by the starch biosynthesis genes mentioned above were actually present in the scutellum, extracts of the scutellum, the embryo and the rest of the germinated grain were subjected to western hybridization analyses (Supplementary Figure S1). Antibodies against the SS1 and SBE1 were available (Cuesta-Seijo et al., 2016) for these analyses and showed that proteins of the expected molecular mass of 68 and 102 kDa, respectively, were present in the embryo and scutellum tissue extracts. Extensive degradation of the enzymes, which might be expected if they originated from the developing grain, was not evident. The apparent increase of both enzymes between 24 and 96 h is consistent with *de novo* synthesis in the germinated grain (Supplementary Figure S1).

**FIGURE 6:**
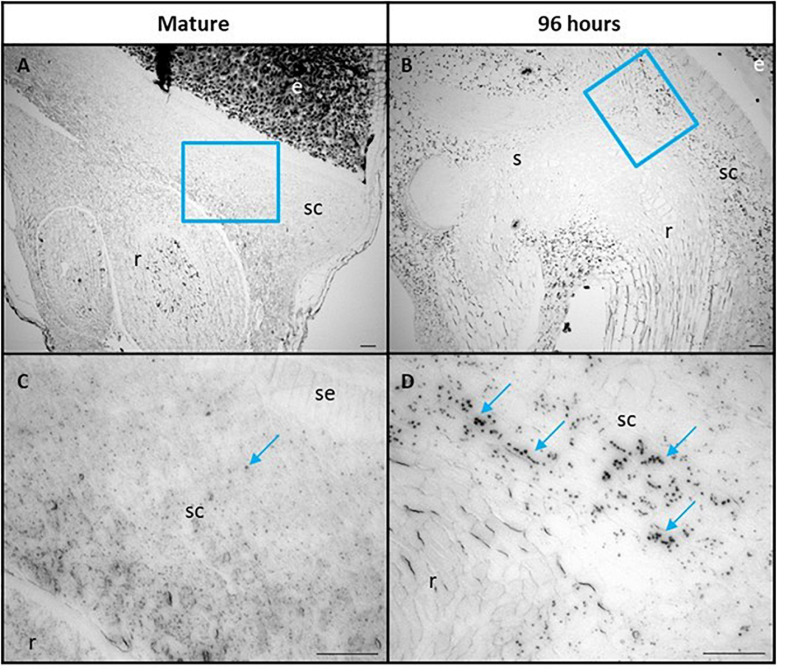
Scutellum cells of mature **(A,C)** and 96 h germinated barley grain **(B,D)** stained with Lugol’s iodine to indicate the presence of starch granules. **(C,D)** are higher magnification images of areas within the blue boxes in **(A,B)**, respectively. Selected starch granules are indicated in **(C,D)** with blue arrows. Scale bar is 50 μm. e, endosperm; sc, scutellum; se, scutellar epithelium; s, shoot; r, root.

The scutellum cells were also examined for the presence of starch granules, using Lugol’s iodine stain (Figure 6). Some starch granules are visible in mature grain at 0 h, particularly in the parenchyma cells of the scutellum (Figures 6A,C), but become much more abundant at 96 h, when they are again more abundant in the parenchyma cells of the scutellum, compared with the scutellum epithelial layer (Figures 6B,D). The starch granules in the scutellum are relatively small, generally about 2–3 μm in diameter.

### Comparison of Branch Lengths of Amylopectin in the Embryo and the Starchy Endosperm

Given the differences in transcript levels of genes involved in starch synthesis in the developing and germinated grain (such as *SBE, GBSS*, and *ISA*; Figure 3A cf. Figure 5) and the observation that different combinations of enzymes can lead to different amylopectin structures (Smith, 2001; Zhong et al., 2020), we investigated the internal chain lengths of this component of the starch granule. Starch was extracted and purified from the scutellum, the embryo and the starchy endosperm of germinated grain and the chain length distributions of the starches were determined following pullulanase/isoamylase hydrolysis and HPAEC-PAD (Botticella et al., 2018). The differences were generally small, although the endosperm starch appeared to have slightly more chains of DP 10–20 than the embryo/scutellum starches, while the scutellum starch at 24 h after the initiation of germination appeared to have more chains in the DP 7–10 range (Supplementary Figure S2). In view of the small differences observed, the fine structures of these amylopectins were not studied further.

### Spatio-Temporal Transcription of Genes Encoding Starch Metabolism Enzymes in Germinated Grain

**FIGURE 7:**
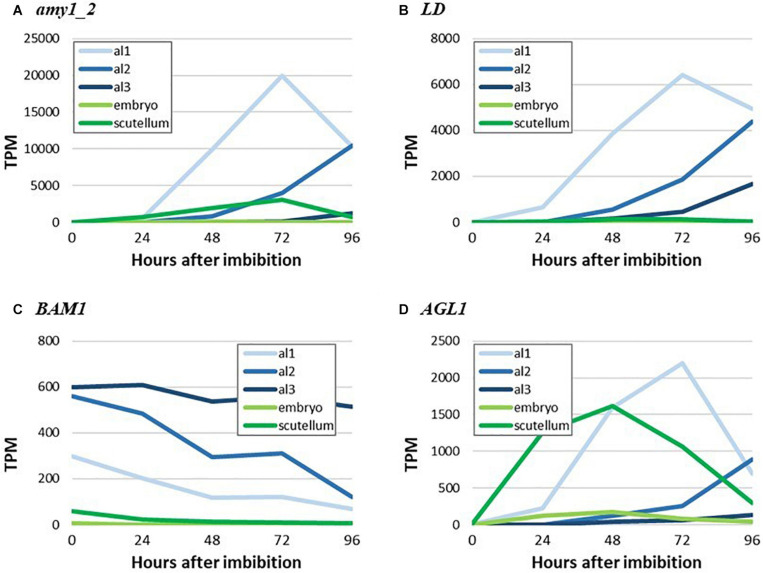
RNA abundance for genes encoding key starch-degrading enzymes in germinated grain. **(A)** *amy_2*, **(B)** *LD*, **(C)** *BAM1*, and **(D)** *AGL1*.

To illustrate more clearly the major findings shown as heat maps in Figures 4, 5, we have extracted TPM data for highly transcribed genes that mediate starch biosynthesis and degradation in germinated grain from Betts et al. (2020) and plotted their tissue and temporal transcription patterns in quantitative terms. Thus, Figure 7 shows the spatiotemporal development of gene transcripts encoding starch degrading enzymes in germinated grain, where *Amy1_2*, *LD* and *AGL1* transcripts peak in abundance at 72 h after the initiation of germination. The progression of transcription from al1 (proximal region of the aleurone) to al3 (distal region of the aleurone) is apparent, although there were also relatively high levels of the *AGL1* transcripts in the scutellum from 0 to 48 h after the initiation of germination. Transcripts of the *Bmy1* gene decreased with time (Figure 7) and are likely, in large part, to reflect residual transcripts from the developing grain, where *Bmy1* transcripts are exceptionally high (Table 1).

**FIGURE 8:**
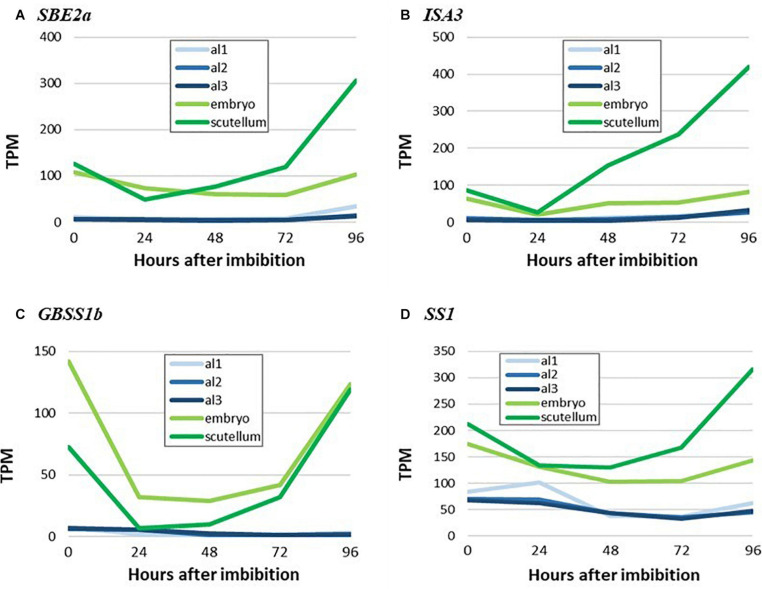
RNA abundance for genes encoding key starch biosynthesis enzymes in germinated grain. **(A)**, *SBE2a*, **(B)** *ISA3*, **(C)** *GBSS1b*, and **(D)** *SS1*. Note the increases in transcripts for starch synthesis genes in the scutellum from 24 to 96 h after imbibition. Embryo denotes the remains of the embryo after removal of the scutellum (Betts et al., 2020), al1 is the proximal aleurone, al2 is the central region of the aleurone and al3 is the dorsal region of the aleurone.

The micrographs presented in Figure 6 indicate that starch is synthesized in the scutellum of the germinated grain. Consistent with this observation, highly transcribed genes involved in starch synthesis (*SBE2a*, *ISA3, GBSS1b*, and *SS1*) are found predominantly in the scutellum of germinated grain (Figure 8). In each case, transcript abundances decrease in the first 24 h after imbibition, but thereafter increase. The transcripts observed at 0 h presumably represent residual mRNA fragments from the developing grain. It should be noted that the ISA3 enzyme could be involved in either or both starch synthesis and degradation in the germinated grain (Figure 1).

**FIGURE 9:**
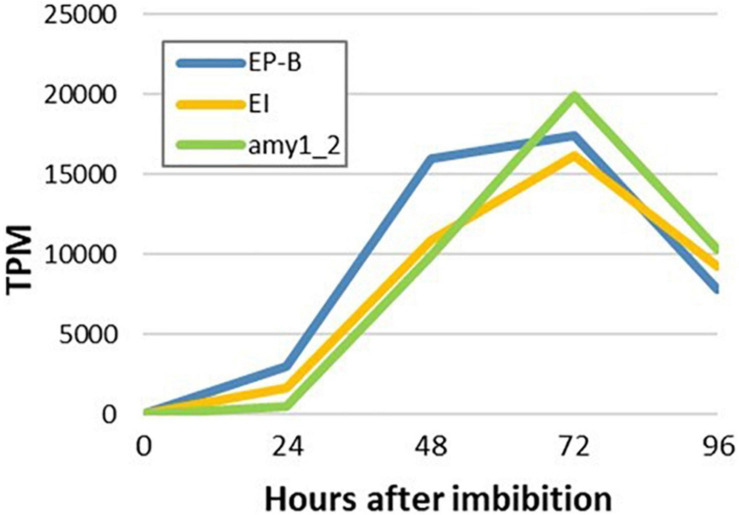
Temporal transcription of genes encoding major enzymes involved in cell wall, starch and storage protein degradation in germinated barley grain. Transcript abundances are from the aleurone al1 tissue extracts. EP-B denotes cysteine endoprotease B, E1 denotes (1,3;1,4)-β-glucanase isoenzyme E1 and Amy1_2 denotes α-amylase Amy1_2.

To detect any temporal differences between the transcription of genes that encode hydrolytic enzymes involved in cell wall, starch and storage protein degradation in the germinated grain, transcript levels of major genes that mediate these processes were investigated (Figure 9). The gene encoding (1,3;1,4)-β-glucanase isoenzyme E1 (HORVU1Hr1G057680) was chosen to represent cell wall degradation, cysteine endoprotease B (HORVU3Hr1G091800) to represent storage protein degradation and *Amy1_2* to represent starch degradation. High transcript levels for these genes were observed and in each case they reached a maximum at 4 days after imbibition (Figure 9).

## Discussion

In the developing barley grain the deposition of starch reserves in the starchy endosperm is a major biochemical activity. Starch synthesis gene transcripts were detected previously by microarray analysis in the very early stages of barley grain cellularization, from 3 to 8 DPA (Zhang et al., 2016) and from 0 to 24 DPA (Radchuk et al., 2009), but here we have used RNA-seq to identify genes involved in starch synthesis between 6 and 42 DPA (Supplementary Table S2).

The transcripts for *GBSS1a* and *GBSS1b* genes, which mediate amylose biosynthesis (Patron et al., 2002) and are located on different chromosomes, are predominantly found in the endosperm and embryo, respectively (Figure 3A and Supplementary Table S2). In contrast, transcripts for the *SS1* and *SS2a* genes predominate amongst the soluble *SS* genes and are found in both the endosperm and embryo (Figure 3A and Supplementary Table S2). These genes mediate the biosynthesis of amylopectin (Denyer et al., 1999). Of the *ISA* genes, which can influence the final internal chain length of amylopectin (Smith, 2001), transcripts for the *ISA3* gene predominate and are detected mainly in the embryo (Supplementary Table S2).

It can be concluded from these data that starch biosynthetic enzymes are expressed in both the embryo and the endosperm of developing barley grain (Table 1). The data allow the identification of key genes that participate in starch synthesis in the different tissues as development proceeds. Differences between expression patterns of individual members of gene families may be important determinants of starch amylose/amylopectin composition and amylopectin fine structures (Nakamura et al., 2005; Zhong et al., 2020) in different tissues and at different times. As mentioned above, our data provide potential target genes for the manipulation of starch content of cereal grains (Gu et al., 2020), relative amylose and amylopectin contents (Zhong et al., 2020), and amylopectin fine structure for nutritional or industrial applications (Blennow, 2018). The *AGP-L1, SBE1*, *SBE2a*, *SBE2b, GBSS1a*, *SS1*, *SS2a*, and *ISA1* genes represent such potential targets, based on the relative abundance of their transcripts during starch synthesis in the developing grain (Table 1). For example, manipulation of expression patterns of the *GBSS1a, GBSS1b, SS1*, and *SS2a* genes could lead to changes in the amylose:amylopectin ratios in starch of the developing starchy endosperm, while manipulation of *SBE1, SBE2a, SBE2b*, and *ISA1* gene expression could be used to alter the internal chain length in amylopectin.

In the context of manipulating the fine structure of amylopectin (Nakamura et al., 2005; Zhong et al., 2020), the chain lengths of amylopectins from the starchy endosperm, scutellum and embryo were examined to identify any major differences in internal chain lengths (Supplementary Figures S2A,B). The peaks for chain length distributions for all samples were similar at DP 11–12, although some differences were observed in the relative abundance of oligosaccharides in the DP ranges of 6–9 and 15–25 (Supplementary Figure S2C). Overall differences were relatively small and we are unable at this stage to correlate transcript levels of particular genes with these small changes in amylopectin structure. However, as noted above we have identified specific members of the *SBE* and *ISA* gene families that might be targeted in the future to alter amylopectin structure (Table 1).

Transcripts of genes that mediate the degradation or the modification of starch were also detected in developing grain, mainly in the embryo. Although *Amy* transcripts are essentially absent, transcripts of starch phosphorylase gene (PHO) were detected. These enzymes catalyze the reversible transfer of glucosyl residues from the non-reducing end of the (1,4)-α-glucan chain to form glucose-1-phosphate (Rathore et al., 2009; Table 1). This suggests that some starch that is synthesized in the embryo is subsequently turned over as the grain fills, but there is no evidence for the degradation of the starch that is deposited in the starchy endosperm. Similarly, the presence of low levels of transcripts for the disproportionating enzyme (DPE2; Table 1) suggests that some modification of starch structure may also be occurring in the embryo of developing grain. The only amylase transcripts detected at significant levels in the developing grain were from the *Bmy1* gene and these were found at very high levels in the endosperm (Table 1 and Figure 4A). This is consistent with early reports that Bmy can account for 1% of total protein in the mature, un-germinated grain. The enzyme has little or no action on intact starch granules and, in the mature grain, is usually bound to other proteins in an inactive form (Hara-Nishimura et al., 1986).

In contrast to the predominance of starch biosynthesis in the developing barley grain, starch degradation is a major activity in the germinated grain. Bound Bmy is released in an active form and acts synergistically with Amy, ISA, LD, and AGL enzymes that are secreted from the aleurone layer to depolymerize starch to small maltodextrins and ultimately to glucose (Figure 1; Fincher, 1989). Four days after the initiation of germination, extensive surface erosion of both large and small starch granules in the starchy endosperm of germinated grain is evident (Figure 4C). The RNA-seq dataset showed that very high transcript levels of the *Amy1_2* gene were expressed in the aleurone layer, with a peak of 19,960 TPM at 72 h in al1 tissue (Table 1). High levels of the *AGL1* gene and the single *LD* gene were also present in the aleurone, again with peaks at 72 h in al1 tissue. *Bmy1* transcripts were present in the aleurone at 0 h and trended downwards in al1 and al2 tissues, with only small changes in al3 tissue during the germination process (Figure 4A). It can be concluded that the *Amy1_2* gene is the most important *Amy* gene for the mediation of starch degradation, at least in cv. Navigator, and that there is little *de novo* transcription of the *Bmy1* gene during germination. Thus, the *Bmy1* transcripts may be residual transcripts from the developing grain although there is a recent report that some Bmy can synthesized *de novo* in germinated barley grain (Vinje et al., 2020). The *Amy1_2* gene, and to a lesser extent the *Amy2_3* and *LD* genes (Table 1), therefore represent candidate genes for targeted alterations of the rate and extent of starch degradation in the germinated grain.

It has been suggested that starch is synthesized transiently in the scutellum during germination, to temporarily store excess glucose that is transported into the scutellum from the rapidly mobilizing starchy endosperm; this transient starch is subsequently degraded and transported to support the growth of the developing seedling (Nieuwdorp and Buys, 1964; Smart and O’Brien, 1979). Consistent with these earlier observations, small starch granules were visible in the parenchyma cells of the scutellum of mature, un-germinated grain and these increased in abundance during germination (Figure 6). The RNA-seq data show that many of the same genes are involved in starch synthesis in the scutellum of germinated grain and in the endosperm of developing grain (e.g., *AGP-L1*, *SBE2a, SS1*, and *GBSS1b*). However, some clear differences are apparent (e.g., levels of *SBE1* transcripts and the relative abundance of *ISA1/ISA3* transcripts Table 1). Similarly, many of the genes involved in starch degradation in the scutellum of germinated grain are also transcribed in the aleurone (e.g., *Amy1_2* and *Amy2_*3; Table 1). To check if genes encoding starch-synthesizing enzymes were being expressed in the germinated grain at the enzyme level, we obtained specific antibodies against the barley SBE1 and SS1 enzymes (Cuesta-Seijo et al., 2016). Western hybridization analyses were performed on grain extracts 24 and 96 h after the initiation of germination. Proteins of the correct size were present in the extracts, their levels appeared to be approximately the same at 24 and 96 h, or perhaps slightly higher at 96 h, and there were relatively low levels of degraded enzyme fragments (Supplementary Figure S1). These protein data are consistent with the presence of their corresponding mRNAs in the RNA-seq analyses (Table 1) and with a role for the two enzymes in the deposition of starch granules in the scutellum of germinated grain (Figure 6).

Morphological studies of germinated cereal grains indicate that there is a clear temporal progression in the mobilization of the starchy endosperm, where degradation of cell walls occurs first, followed by the removal of storage protein and finally in the erosion of starch granules (Fincher, 1989). To further investigate these observations, temporal patterns of (1,3;1,4)-β-glucanase isoenzyme E1 (Slakeski and Fincher, 1992), cysteine endoprotease B (Koehler and Ho, 1990; Marttila et al., 1995) and Amy1_2 (Radchuk et al., 2009) transcript abundances were compared (Figure 9). However, no clear differences in the temporal patterns of transcript abundances were apparent (Figure 9), although differences might be occurring within the first 24 h after the initiation of germination.

## Conclusion

In summary, we have isolated individual tissues from intact developing barley grain from 6 to 42 DPA to generate transcript profiles for genes that mediate both starch synthesis and degradation. These data were compared to a previous database for dissected tissues from germinated barley grain (Betts et al., 2020). Complete RNA-seq transcript datasets for 81,280 genes in developing grain (Supplementary Table S2) and 33,421 genes in germinated grain (Betts et al., 2020), together with their HORVU identifiers, are publicly available and can be mined for genes that mediate other important biochemical and cellular processes in developing and/or germinated grain. Transcript profiles of genes involved in both starch synthesis and depolymerization have allowed a detailed spatio-temporal picture of starch metabolism to be defined not only in developing grain from pollination through to maturity, but also in individual tissues of germinated grain. Our identification here of key members of the large gene families that mediate starch synthesis and degradation in developing and germinated grain can now be exploited to optimize or manipulate the content, composition and fine structure of starch components in cereal grains for food, human health and industrial applications, through GMO technologies or through mining gene variants in germplasm, breeding or induced mutant populations.

## Data Availability Statement

RNA-seq data was deposited at the NCBI SRA database under project IDs PRJNA533515 (germinated grain) and PRJNA680168 (developing grain).

## Author Contributions

BS, GF, VB, and JW conceived and designed the research project. HC, NB, CD, OB, JC-S, and IB performed the experiments and analyzed the data. GF was responsible for preparing the manuscript, with contributions from the other authors. All authors read and approved the final manuscript.

## Conflict of Interest

CD, IB, JC-S, and BS were employed by the Carlsberg Research Laboratory within the Carlsberg Group. The remaining authors declare that the research was conducted in the absence of any commercial or financial relationships that could be construed as a potential conflict of interest. The authors declare that this study received funding from the Carlsberg Foundation and Coopers Brewery. The funders were involved in some elements of the experimental design data collection and analysis, decision to publish, and preparation of the manuscript.

## Footnotes

1 http://www.illumina.com/

## Supplementary Material

Supplementary Figure 1 Western blots probed with starch enzyme antibodies.

Supplementary Figure 2 Amylopectin chain lengths in starch from different tissues.

Supplementary Table 1 List of genes with respective HORVU IDs (Mascher et al., 2017) involved in starch metabolism.

Supplementary Table 2 Excel spread sheet of TPM values for transcripts of 81,280 genes transcribed in the developing barley grain.
