# Supplementary material for: Genes That Mediate Starch Metabolism in Developing and Germinated Barley Grain
Source: Front Plant Sci. 2021 Mar 1;12:641325. doi: 10.3389/fpls.2021.641325 (PMC7959180; doi:10.3389/fpls.2021.641325)

SUPPLEMENTARY FIGURE S1 Western blots probed with starch enzyme antibodies. (A) starch synthase SS1. **(B)** starch branching enzyme SBE1. E, embryo; S, scutellum; R, rest of grain; M, marker; Rec, recombinant protein control. Numbers in lane labels refer to germination time, i.e., S24 is scutellum tissue 24 h into germination. Numbers on the sides of the gel give kDa masses for marker bands and bands of interest.

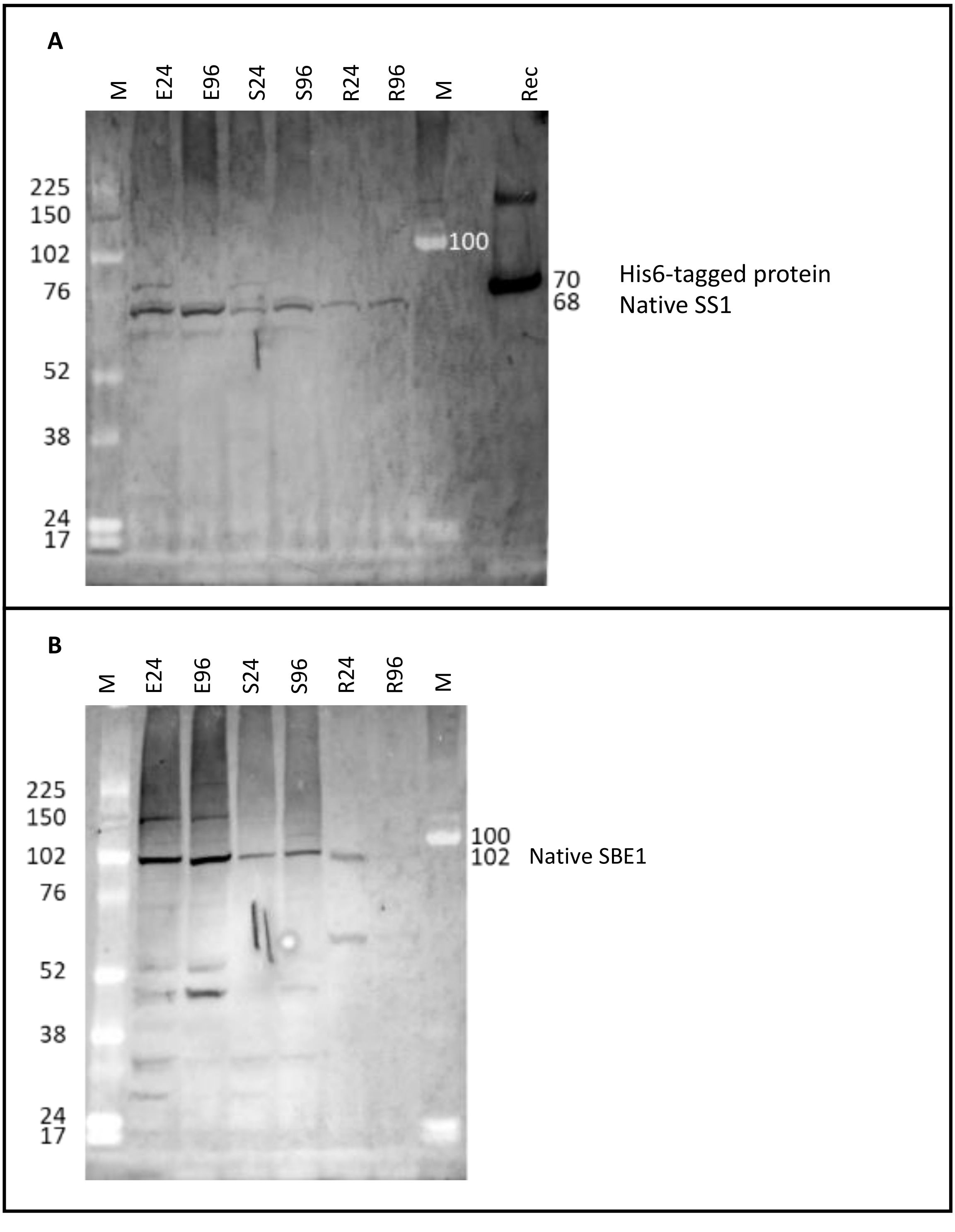

Supplement: Supplementary Figure 1 — Western blots probed with starch enzyme antibodies. [file Image_1.pdf]
