# Supplementary material for: Genes That Mediate Starch Metabolism in Developing and Germinated Barley Grain
Source: Front Plant Sci. 2021 Mar 1;12:641325. doi: 10.3389/fpls.2021.641325 (PMC7959180; doi:10.3389/fpls.2021.641325)

SUPPLEMENTARY FIGURE S2 Amylopectin chain lengths in starch from different tissues. (A) Starch from scutellum at 24 h and 96 h compared with endosperm starch. **(B)** Starch from embryo at 24 h and 96 h compared with endosperm starch. **(C)** Normalized peak areas (%) for oligosaccharides with degrees of polymerisation from 2 to 60, in all samples.

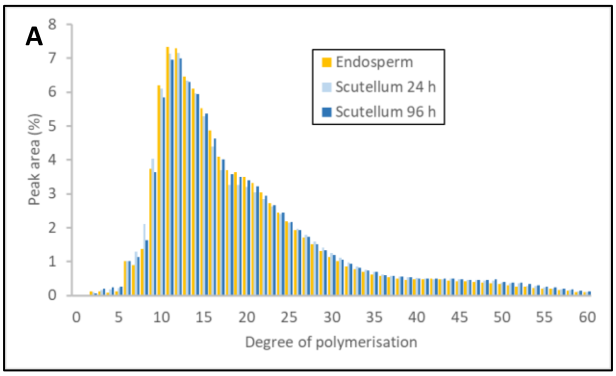

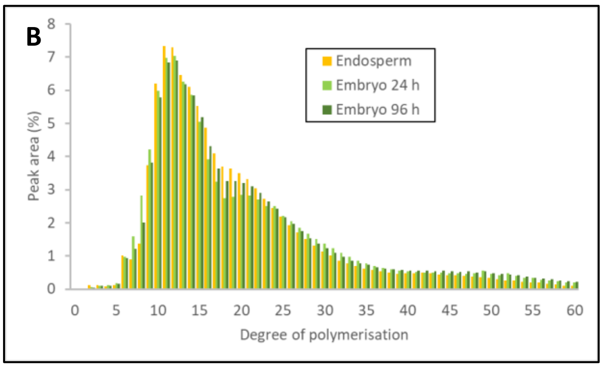

C

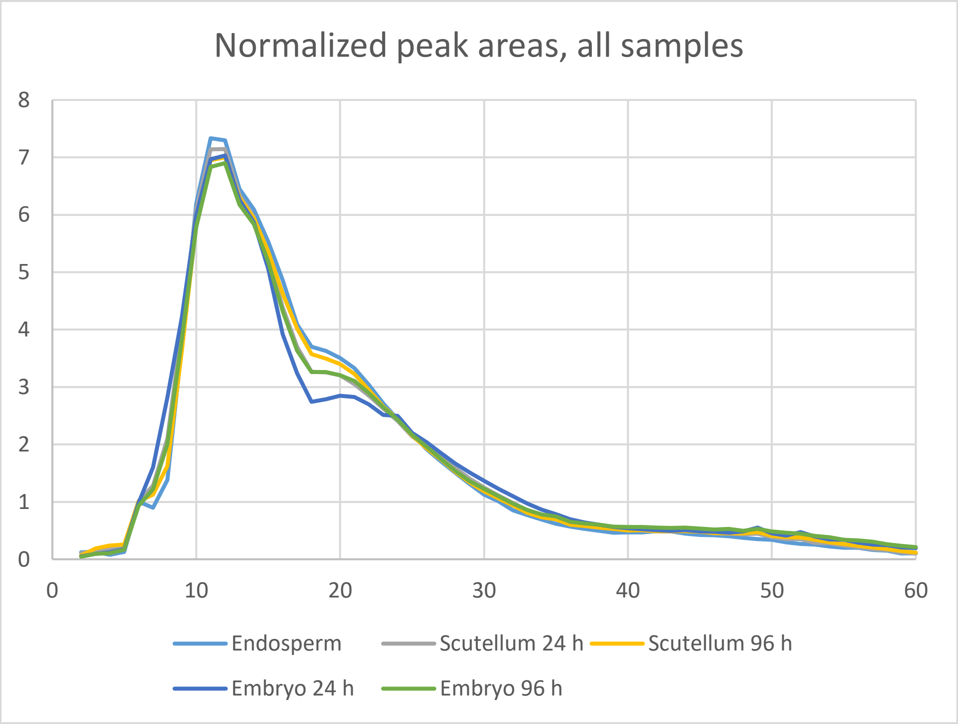

Supplement: Supplementary Figure 2 — Amylopectin chain lengths in starch from different tissues. [file Image_2.pdf]
