# Supplementary material for: Genes That Mediate Starch Metabolism in Developing and Germinated Barley Grain
Source: Front Plant Sci. 2021 Mar 1;12:641325. doi: 10.3389/fpls.2021.641325 (PMC7959180; doi:10.3389/fpls.2021.641325)
Supplement: Supplementary Table 1 — List of genes with respective HORVU IDs (Mascher et al., 2017) involved in starch metabolism. [file Table_1.docx]

**Supplementary Table S1.** List of genes with respective HORVU IDs (Mascher et al., 2017) involved in starch metabolism**

 Note: Transcripts from two fragments of the AGP-L2 gene, both of which showed similar developmental profiles, were detected but only the higher expressing fragment is included in our analyses.
